# Effectiveness of Bisphosphonate Analogues and Functional Electrical Stimulation on Attenuating Post-Injury Osteoporosis in Spinal Cord Injury Patients- a Systematic Review and Meta-Analysis

**DOI:** 10.1371/journal.pone.0081124

**Published:** 2013-11-22

**Authors:** Ke-Vin Chang, Chen-Yu Hung, Wen-Shiang Chen, Mei-Shu Lai, Kuo-Liong Chien, Der-Sheng Han

**Affiliations:** 1 Department of Physical Medicine and Rehabilitation, National Taiwan University Hospital, BeiHu Branch and National Taiwan University College of Medicine, Taipei, Taiwan; 2 Graduate Institute of Epidemiology and Preventive Medicine, National Taiwan University, Taipei, Taiwan; 3 Department of Physical Medicine and Rehabilitation, National Taiwan University Hospital and National Taiwan University College of Medicine, Taipei, Taiwan; 4 Department of Internal Medicine, National Taiwan University Hospital and National Taiwan University College of Medicine, Taipei, Taiwan; UCLA, United States of America

## Abstract

**Background:**

Various pharmacologic and non-pharmacologic approaches have been applied to reduce sublesional bone loss after spinal cord injury (SCI), and the results are inconsistent across the studies. The objective of this meta-analysis was to investigate whether the two most-studied interventions, bisphosphonate analogues and functional electrical stimulation (FES), could effectively decrease bone mineral density (BMD) attenuation and/or restore lost BMD in the SCI population.

**Methods:**

Randomized controlled trials, quasi-experimental studies, and prospective follow-up studies employing bisphosphonates or FES to treat post-SCI osteoporosis were identified in PubMed and Scopus. The primary outcome was the percentage of BMD change from baseline measured by dual-energy X-ray absorptiometry (DEXA) or computed tomography (CT). Data were extracted from four points: the 3rd, 6th, 12th, and 18th month after intervention.

**Results:**

A total of 19 studies were included in the analysis and involved 364 patients and 14 healthy individuals. Acute SCI participants treated with bisphosphonate therapy demonstrated a trend toward less bone loss than participants who received placebos or usual care. A significant difference in BMD decline was noted between both groups at the 3rd and 12th month post-medication. The subgroup analysis failed to show the superiority of intravenous bisphosphonate over oral administration. Regarding FES training, chronic SCI patients had 5.96% (95% CI, 2.08% to 9.84%), 7.21% (95%CI, 1.79% to 12.62%), and 9.56% (95% CI, 2.86% to 16.26%) increases in BMD at the 3rd, 6th, and 12th months post-treatment, respectively. The studies employing FES ≥5 days per week were likely to have better effectiveness than studies using FES ≤3 days per week.

**Conclusions:**

Our meta-analysis indicated bisphosphonate administration early following SCI effectively attenuated sublesional bone loss. FES intervention for chronic SCI patients could significantly increase sublesional BMD near the site of maximal mechanical loading.

## Introduction

Substantial reductions in bone mineral density (BMD) are inevitable below the level of the lesion in spinal cord injury (SCI) patients. Rapid bone loss occurs in the early months following the injury and continues for more than two years until a steady state has been reached [1]. Marked increases in osteoclastic bone resorption and decreases in osteoblastic bone formation likely occur as a result of immobilization, non-weight bearing, and dysregulation of neuroendocrine systems [2,3]. The disruption of the normal skeletal metabolism places the weight bearing bones, such as the femur and proximal tibia, at a greater risk of fragility fractures [2]. Most fractures in the SCI population result from minor trauma and can easily lead to complications, such as breakdown of skin integrity, infection, and fracture malunion, all of which additionally decrease the quality of life and increase healthcare costs. Therefore, an intervention that can effectively attenuate or reverse the bone loss would be beneficial for all SCI patients.

Various treatments including medication, exercise, and physical modalities have been applied to SCI patients to investigate the efficacy of reducing sublesional BMD attenuation [4-8]. Regarding pharmacologic treatments, bisphosphonates emerge as the most-studied regimen and have been tested in paraplegic patients since 1981 [9]. Although bisphosphonate analogues are recognized as effective antiresorptive drugs to prevent osteoporotic fractures in postmenopausal women [10], their role in maintaining BMD following SCI remains controversial [8]. The inconsistent results across the bisphosphonate studies might be derived from various timings and routes of administration, different potencies of analogues, heterogeneity of enrolled patients, and research methodology. In terms of non-pharmacologic therapies, weight bearing, electrical stimulation with or without functional tasks, vibration, pulsed electromagnetic fields, and ultrasound modalities have been evaluated for their effects on modifying BMD in SCI participants [7]. Functional electrical stimulation (FES) is the most extensively investigated approach since it combines the benefits from electrical stimulation and mechanical loading. Although FES intervention has been demonstrated to improve muscle atrophy, solid evidence from a large scale study is still lacking regarding its influence on sublesional BMD attenuation. To our knowledge, a quantitative analysis of BMD changes following either bisphosphonate administration or FES training has never been performed in a specific patient group. The present meta-analysis would aim to investigate the usefulness of both interventions against osteoporosis after SCI and explore whether the effectiveness might vary based on differences in regimens and timing of administration.

## Materials and Methods

### Study selection

A systematic review was performed by searching PubMed and Scopus from the earliest records to January of 2013. SCI, paraplegia, quadriplegia, BMD, osteoporosis, and osteopenia were entered as medical subject headings and text words to identify relevant articles. Bisphosphonate, alendronate, zoledronic acid, pamidronate, functional electrical stimulation, bicycle, cycling, and exercise were key terms used to extract studies using bisphosphonate analogues or FES to attenuate bone loss. Cochrane Collaboration Central Register of Controlled Clinical Trials, Cochrane Systematic Reviews, ClinicalTrials.gov and bibliographies of included trials and related systematic reviews or meta-analyses were manually scrutinized for additional references.

### Eligibility criteria

Randomized controlled trials, quasi-experimental studies, and prospective follow-up studies were included in the review without language restriction. Case reports or case series without a well-designed intervention scheme or outcome measurement were excluded. Studies were eligible if they enrolled adult participants with traumatic SCI. Trials presenting data on pediatric participants and people with specific causes for SCI such as infection, neoplasm, inflammatory diseases, and vasculopathy were ruled out. The interventions of included articles were limited to administration of bisphosphonate analogues and FES. FES was defined as a technique that uses electrical currents to activate nerves innervating paralytic extremities to perform a functional task such as bicycling, ambulation, or resistance training. Research was eliminated if it merely employed electric currents to stimulate paralytic limbs without concomitant exercise regimens. All the selected trials were required to have BMD measured by dual-energy X-ray absorptiometry (DEXA) or a computed tomography (CT).

### Data extraction and quality assessment

Two authors (K.V.C. and C.Y.H.) independently evaluated all potential articles eligible for inclusion. Data extracted from selected trials included study designs, patient characteristics, features of bisphosphonate or FES administration, and details of outcome measurements. The Jadad scale was used to assess the quality of the randomized controlled or quasi-experimental trials. The aggregate scores ranged from 0 to 5 points [11]. Trials with scores <3 were assumed to have lower methodologic quality. Prospective cohort or longitudinal follow-up studies were evaluated using the Newcastle-Ottawa scale to assess the quality of selection, comparability, exposure, and outcome [12]. The maximum scores observed were 9 points, and total scores <4 points were considered low in quality. Discrepancies between the 2 independent evaluations of potential articles were resolved by discussion and consensus.

### Data synthesis and statistical analysis

Data were extracted from four points: the 3rd, 6th, 12th, and 18th month after intervention. The primary outcome was the percentage of BMD change from baseline and standard deviation (SD) as measured by DEXA or CT. If it was not directly reported, the percentage of BMD change was calculated from the difference of BMD between baseline and follow-up divided by BMD at baseline. The standard deviation was estimated from the square root [(SD of mean BMD at baseline)^2^ + (SD of BMD during follow-up)^2^- (SD of BMD at baseline) * (SD of BMD during follow-up)] divided by BMD at baseline [13,14]. The distal femur was the main site used to calculate the BMD. If this data was not reported, the BMD measured at the proximal tibia followed by the femoral trochanter, femoral neck, and the whole lower extremity were utilized instead. In order to maintain comparability with data recorded by DEXA, total BMD rather than trabecular BMD data from CT measurements was used for analysis.

The random effect model was employed to provide a relatively conservative estimate with a 95% confidence interval (CI) of the pooled percent changes in BMD [15]. The heterogeneity across studies was tested by I square and Cochran’s Q test. A P value <0.1 for chi-squared testing of the Q statistic or an I square >50% was regarded as the existence of significant heterogeneity [15]. We performed a subgroup analysis according to the post-injury duration, route of bisphosphonate administration, and the frequency of FES training. A post-injury duration less than two years was defined as acute SCI, whereas chronic SCI indicated patients were undergoing treatments beyond two years following injury. Regarding the acute SCI group, a superiority of intervention was determined by a lower BMD decline in the intervention group without an overlap of the 95% CI of the controls. Regarding the chronic SCI group, we assumed that the BMD attenuation had reached a steady state, and an advantage of intervention referred to an increase in BMD from baseline with a 95% CI above the value of zero. All analyses were performed using Stata 10.0 (StataCorp, Texas, USA).

## Results

**Figure 1:**
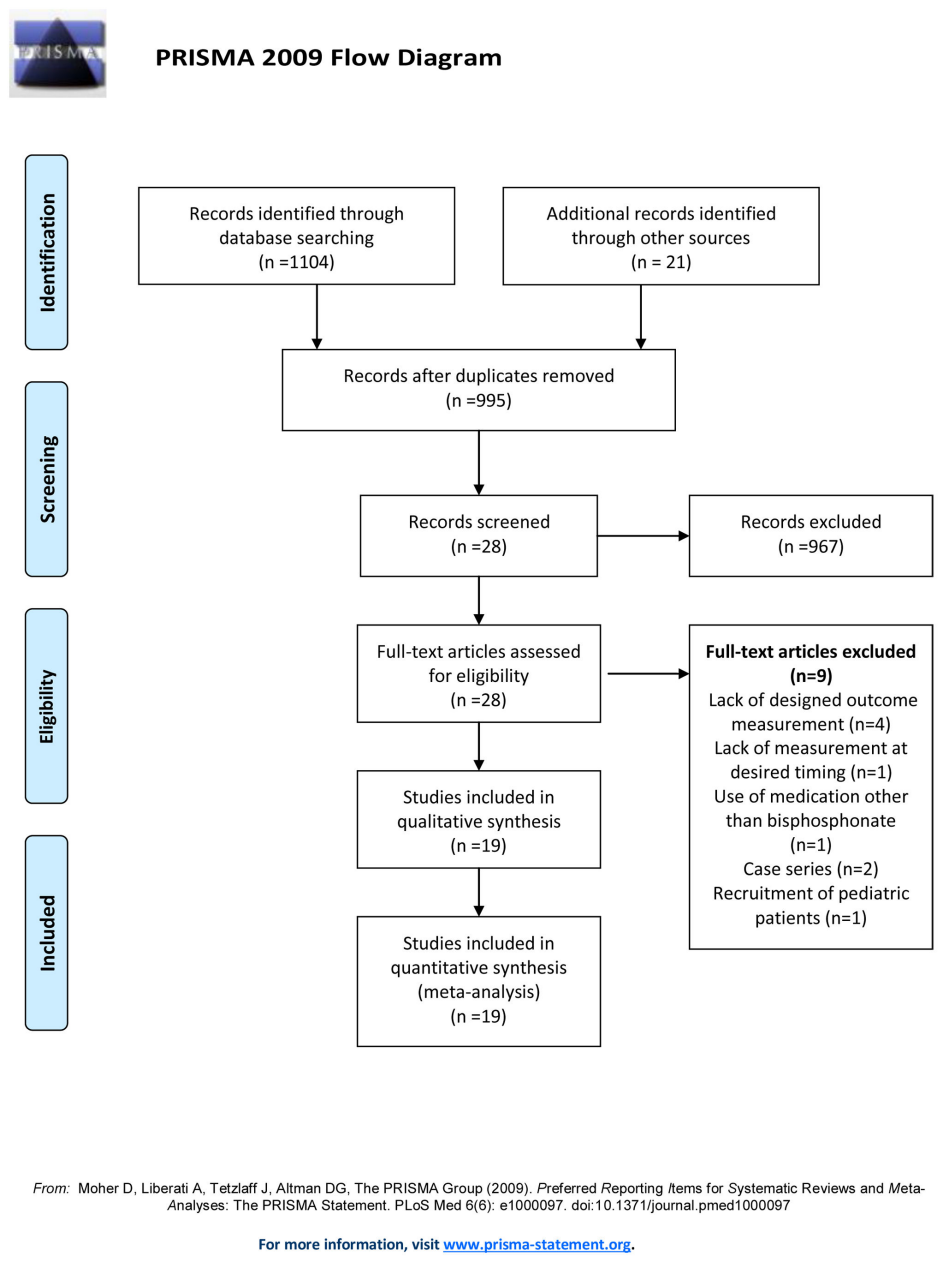
Flow diagram of the evaluation process for the included and excluded studies.

Of the 28 non-duplicate citations identified from the literature, 12 clinical trials associated with bisphosphonate usage and 16 associated with FES administration were screened for eligibility (Figure 1). Four studies were excluded due to medication use other than bisphosphonate analogues [16] or lack of an outcome measured by a DEXA or CT [9,17,18]. An assessment of the remaining 8 articles revealed 7 randomized controlled trials [19-25] and one quasi-experimental study [26]. Among the 7 randomized controlled trials, 3 employed the double-blind placebo controlled scheme [21,23,24]. With regard to bisphosphonate regimens, three studies [20,22,23] used oral alendronate, one study [19] used oral etidronate, two studies [24,25] used intravenous (IV) zoledronic acid, and two studies [21,26] used IV pamidronate. With respect to reference treatments, five studies [19,20,25,26] utilized usual care, two studies [21,24] utilized normal saline injections, and one study [23] utilized placebo tablets.

With respect to FES training, 5 studies were excluded: two studies [27,28] were case series without pre-designed outcome measurements; one study [29] did not report BMD at our desired timing; one study [30] was deficient in BMD assessment; and another study [31] employed pediatric SCI patients. Among the 11 enrolled articles, 9 articles [32-41] were longitudinal follow-up cohort studies whose effectiveness of intervention was determined by the comparison with baseline, and two studies [32,33] used quasi-experimental designs which enrolled SCI participants with usual care. None were randomized controlled trials. In terms of the FES regimens, nine studies [32-34,36,38-42] employed FES cycling ergometry, and 2 studies [35,37] employed FES plus resistance training.

### Characteristics of included patients (Table 1)

**Table 1:** Summary of studies which used bisphosphonate analogues or functional electrical stimulation (FES) to treat bone mineral density (BMD) loss in patients after spinal cord injury (SCI).

**Authors, year**	**Sample characteristic**	**Sample number**	**Study design**	**Double blind**	**Intention to treat**	**Treatment**	**Frequency**	**Duration**	**Device for BMD measurement**	**Site of BMD measurement**	**Quality Assessments**
**Studies using bisphosphonate analogues to attenuate bone loss in SCI patients**											
Pearson et al, 1997	Acute SCI. Post-injury duration: within 6 weeks. Injury level: T: C5-T12; C: C6-T12. Age: T: 33.6 ± 4.5 years; C: 35.6 ± 11.6 years	T: 5 (4 males, 1 female); C: 6 (all males)	RCT	No	No	Etidronate, 800 mg, oral	Once daily	2 weeks for each cycle; total two cycles of treatments separated by 13 weeks	DEXA	Distal femur	1*
Nance et al, 1999	Acute SCI. Post-injury duration: within 6 weeks. Injury level: T: C4-L1; C: C6-L1. Age: T: 31.8 ± 8.7 years; C: 34.4 ± 12.1 years	T: 12 (11 males, 1 female); C: 7 (all males)	Quasi-experimental study	No	No	Pamidronate, 30 mg, IV	Once per month	6 months	DEXA	Whole lower extremity	0*
Zehnder et al, 2004	Mainly chronic SCI. Post-injury duration: T: 10.8 ± 1.4 years; C: 9.9 ± 1.7 years. Injury level: T1-L3. Age: T: 38.8 ± 1.5 years; C: 37.9 ± 2.2 years	T: 29 (all males); C: 26 (all males)	RCT	No	No	Alendronate, 10 mg, oral	Once daily	24 months	DEXA	Proximal tibia	2*
Moran et al, 2005	Chronic SCI. Post-injury duration: T: 61.0 ± 77.3 months; C: 38.7 ± 17.1 months. Injury level: not mentioned. Age: T: 30.9 ± 9.5 years; C: 30.8 ± 9.9 years	T: 9 (1 dropout from 8 males, 2 females); C: 8 (1 dropout from 7 males, 2 females)	RCT	No	No	Alendronate,10 mg, oral	Once daily	6 month	DEXA	Whole lower extremity	1*
Bauman et al, 2005	Acute SCI. Post-injury duration: 44 ± 18 days. Injury level: T: 3 tetraplegia, 3 paraplegia; C: 2 tetraplegia, 3 paraplegia. Age: T: 39 ± 15 years; C: 30 ± 8 years	T: 6 (4 males, 2 females); C: 5 (4 males, 1 female)	RCT	Yes	No	Pamidronate, 60 mg IV	at baseline and then at 1, 2, 3, 6, 9, and 12 months	12 month	DEXA	Distal femur	4*
Gilchrist et al, 2007	Acute SCI. Post-injury duration: within 10 days. Injury level: C4-L2. Age: 17-55 years	T: 12 (3 dropouts from 10 males, 5 females); C: 13 (3 dropouts from 12 males, 4 females)	RCT	Yes	No	Alendronate, 70 mg, oral	Once per week	12 months	DEXA	Femoral shaft	5*
Shapiro et al, 2007	Acute SCI. Post-injury duration: < 12 weeks. Injury level: C2-T12. Age: T: 30.1 ± 14.2 years; C: 28.4 ± 9.4 years	T: 8; C: 9 (gender not mentioned)	RCT	Yes	No	Zoledronic acid, 4 or 5 mg, IV	At baseline	Once	DEXA	Femoral neck	5*
Bubbear et al, 2011	Acute SCI. Post-injury duration: within 3 months. Injury level: T: C4-L3; C: C6-T8. Age: 31.6 ± 7.7 years; C: 27.0 ± 14.4 years	T: 6 (1 dropout from 4 males, 3 females); C: 5 (2 dropouts from 5 males, 2 females)	RCT	No	No	Zoledronic acid, 4mg, IV	At baseline	Once	DEXA	Greater trochanter	3*
**Studies using FES to attenuate bone loss in SCI patients**											
Leeds et al, 1990	Chronic SCI. Post-injury duration: 5.17 ± 2.40 years. Injury level: C4-C6. Age: 23.67 ± 3.20 years	6 (all males)	Longitudinal follow-up study	No	Yes	FES cycling ergometry	3 sessions per week. Gradually increased training time to 30 mins per session	6 months	DEXA	Femoral trochanter	4†
BeDell et al, 1996	Chronic SCI. Post-injury duration: > 2 years. Injury level: C5-T12. Age: 34 ± 6 years	12 (all males)	Longitudinal follow-up study	No	Yes	FES cycling ergometry	30 mins, three times per week	24 sessions	DEXA	Femoral neck	4†
Bloomfield et al, 1996	Chronic SCI Post-injury duration: T: 6 ± 1.2 years; C: 8.3 ± 2.3 years. Injury level: T: C5-T7; C: C4-T12. Age: T: 28.2 ± 1.8 years; C: 34.4 ± 2.5 years	T: 9 (5 males, 4 females); C: 8 (5 males, 3 females)	Quasi-experimental study	No	Yes	FES cycling ergometry	3 sessions per week	9 months	DEXA	Distal femur	5*
Mohr et al, 1997	Chronic SCI. Post-injury duration: 12.5 ± 2.7 years. Injury level: C6-T4. Age: 35.3 ± 2.3 years	10 (8 males, 2 females)	Longitudinal follow-up study	No	Yes	FES cycling ergometry	30 mins per day, 3 days per week	12 months	DEXA	Proximal tibia	4†
Belanger et al, 2000	Chronic SCI Post-injury duration: 9.6 ± 6.6 years. Injury level: C5-T5. Age: 32.4 ± 5.9 years	T: 14 (11 males, 3 females); C: 14 age and sex-matched healthy individuals	Longitudinal follow-up study	No	No	FES plus resistive training	1 hour per day, 5 days per week	6 months	DEXA	Distal femur	4†
Eser et al, 2003	Acute SCI. Post-injury duration: T: 4.5 ± 2.9 weeks; C: 4.6 ± 2.9 weeks. Injury level: T: C5-T10; C: C5-T12. Age: T: 32.9 ± 11.5 years; C: 33.8 ± 13.0 years	T: 19 (17 males, 2 females) ; C: 19 (17 males, 2 females)	Quasi-experimental study	No	No	FES cycling ergometry	30 mins per day, 3 days per week	Average 6 months	CT scanner	Proximal tibia	5*
Chen et al, 2005	Chronic SCI. Post-injury duration: > 2 years and 7 months. Injury level: C5-T8. Age: 28.67 ± 3.77 years	15 (all males)	Longitudinal follow-up study	No	Yes	FES cycling ergometry	30 mins per day, five days per week	6 months	DEXA	Distal femur	4†
Clark et al, 2007	Acute SCI. Post-injury duration: within two days. Injury level: T: C4-T10; C: C5-T12. Age: T: 30.0 ± 8.9 years; C: 34.8 ± 11.2 years	T: 23; C: 10 (gender not mentioned)	Longitudinal follow-up study	No	Yes	FES plus resistive training	15-min to each leg twice daily, 5 days per week	5 months	DEXA	Whole lower extremity	5†
Frotzler et al, 2008	Chronic SCI. Post-injury duration: 11.0 ± 7.1 years. Injury level: T3-T9. Age: 41.9 ± 7.5 years	11 (9 males, 2 females)	Longitudinal follow-up study	No	No	FES cycling ergometry	60 mins per session. 5 sessions per week	12 months	Peripheral quantitative CT scanner	Distal femur	4†
Griffin et al, 2009	Chronic SCI. Post-injury duration: 11.0 ± 3.1 years. Injury level: C4-T7. Age: 40.0 ± 2.4 years	18 (13 males, 5 females)	Longitudinal follow-up study	No	Yes	FES cycling ergometry	2-3 times per week	10 weeks	DEXA	Not mentioned	4†
Lai et al, 2010	Acute SCI. Post-injury duration: 26-52 days. Injury level: C5-T10. Age: T: 28.9 ± 5.3 years; C: 28.2 ± 5.7 years	T: 12 (10 males, 2 females); C: 12 (10 males, 2 females)	Longitudinal follow-up study	No	Yes	FES cycling ergometry	3 times per week	3 months, and then suspend for subsequent 3 months	DEXA	Distal femur	5†

Note: * Quality scores derived from the Jadad scale. † Quality scores derived from the Newcastle-Ottawa Scale. Abbreviation: T, treatment group; C, control group; RCT: randomized controlled trial; DEXA: dual-energy X-ray absorptiometry; IV: intravenous.

Our meta-analysis included 364 SCI patients and 14 healthy individuals. The majority were men with ages ranging from 23.7 to 41.9 years. The level of the spinal cord lesion varied from C2 to T12, and most of the studies included patients with both complete and incomplete neurologic injury. Regarding the timing of bisphosphonate or FES administration, nine studies [19,21,23-26,37,40,42] enrolled patients with acute SCI, and ten trials [20,22,32-36,38,39,41] recruited mainly chronic SCI participants.

### Percentage of BMD changes (Table 2)

**Table 2:** Percent of bone mineral density (BMD) changes compared with the baseline value at the 3^rd^, 6^th^, 12^th^ and 18^th^ or more in spinal cord injury (SCI) patients after intervention.

**Authors, year**	**Participants’ pattern**	**Intervention**	**Comparator**	**Percent of BMD changes at the 3rd month**	**Percent of BMD changes at the 6th month**	**Percent of BMD changes at the 12th month**	**Percent of BMD changes at the 18th month or more**
**Studies using bisphosphonate analogues to attenuate bone loss in SCI patients**							
Pearson et a al, 1997	Acute SCI	Etidronate (oral)	SCI patients with usual care	No measurement	T: -8.3 ± 3.6; C: difficulty in data extraction	T: -26.1 ± 7.4; C: difficulty in data extraction	No measurement
Nance et al, 1999	Acute SCI	Pamidronate (IV)	SCI patients with usual care	No measurement	No measurement	T: -4.7 ± 0.4; C: -10.8 ± 0.4	No measurement
Zehnder et al, 2004	Mainly chronic SCI	Alendronate (oral)	SCI patients with usual care	No measurement	T: 0.4 ± 0.6; C: -1.1 ± 0.4	T: -0.7 ± 0.8; C: -2.3 ± 0.6	T: -1.3 ± 1.3; C: -4.0 ± 0.82
Moran et al, 2005	Chronic SCI	Alendronate (oral)	Calcium 1000mg daily	No measurement	T: -1.0 ± 1.9; C: -0.9 ± 4.6	No measurement	No measurement
Bauman et al, 2005	Acute SCI	Pamidronate (IV)	Normal saline	T: -1.0 ± 3.0; C: -6.0 ± 7.0	T: -5.0 ± 4.0; C: -9.0 ± 8.0	T: -9.0 ± 7.0; C: -12 ± 7.0	T : -18.0 ± 9.0; C: -19.0 ± 9.0
Gilchrist et al, 2007	Acute SCI	Alendronate (oral)	Placebo tablet	T: -0.3 ± 5.2; C: -5.6 ± 4.7	T: -2.3 ± 5.4; C: -13.4 ± 4.9	T: -3.4 ± 5.2; C: -18.5 ± 4.7	T: -7.1 ± 4.7; C: -22.6 ± 4.3
Shapiro et al, 2007	Acute SCI	Zoledronic acid (IV)	Normal saline	No measurement	T: 2.4 ± 4.3; C: -1.7 ± 3.5	T: -2.1 ± 4.2; C: -12.6 ± 5.2	No measurement
Bubbear et al, 2011	Acute SCI	Zoledronic acid (IV)	SCI patients with usual care	T: -1.9 ± 2.41; C: -10.84 ± 1.72	T: -1.5 ± 5.9; C: -18.5 ± 5.9	T: -4.5 ± 5.7; C: -17.9 ± 9.4	No measurement
**Studies using functional electrical stimulation (FES) to attenuate bone loss in SCI patients**							
Leeds et al, 1990	Chronic SCI	FES cycling ergometry	Nil	No measurement	T: -5.6 ± 6.5	No measurement	No measurement
Bedell et al, 1996	Chronic SCI	FES cycling ergometry	Ni;	T: 5.1 ± 17	No measurement	No measurement	No measurement
Bloomfield et al, 1996	Chronic SCI	FES cycling ergometry	SCI patients with usual care	T: 6.7 ± 2.1; C:-2.1 ± 3.4	T: 4.8 ± 3.3; C: 2.3 ± 4.4	No measurement	No measurement
Mohr et al, 1997	Chronic SCI	FES cycling ergometry	Nil	No measurement	No measurement	T: 9.7 ± 3.5	T: -2 ± 6.9
Belanger et al, 2000	Chronic SCI	FES plus resistive training	Nil	No measurement	T: 11.1 ± 4.6	No measurement	No measurement
Eser et al, 2003	Acute SCI	FES cycling ergometry	SCI patients with usual care	T: -0.9 ± 1.8; C: -2.1 ± 2.4	T: -1.8 ± 3.6; C: -4.2 ± 4.8	No measurement	No measurement
Chen et al, 2005	Chronic SCI	FES cycling ergometry	Nil	No measuremnt	T: 11.1 ± 0.8	No measurement	No measurement
Clark et al, 2007	Acute SCI	FES to quadriceps femoris and anterior tibialis	SCI patients with usual care	T: -2.4 ± 3.3; C: -2.3 ± 2.8	T: -7.1 ± 3.1; C: -4.7 ± 2.7	No measurement	No measurement
Frotzler et al, 2008	Chronic SCI	FES cycling ergometry	Nil	No measurement	T: 5.2 ± 15.6	T: 6.6 ± 16	No measurement
Griffin et al, 2009	Chronic SCI	FES cycling ergometry	Nil	T: -0.6 ± 6.3	No measurement	No measurement	No measurement
Lai et al, 2010	Acute SCI	FES cycling ergometry	Nil	T: -2.1 ± 0.9; C: -6.6 ± 0.5	No measurement	No measurement	No measurement

Note: abbreviation: T: treatment group; C: control group; IV: intravenous

**Figure 2:**
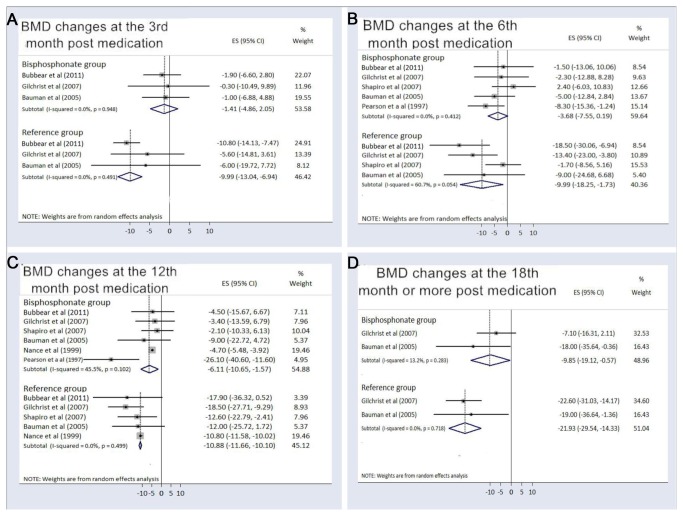
Percent bone mineral density changes in acute spinal cord injury patients using bisphosphonates. The figure represents the forest plot of percent bone mineral density (BMD) changes from baseline in the acute spinal cord injury patients using bisphosphonates compared to the reference group at (A) the 3rd, (B) the 6th, (C) the 12th and (D) the 18th month or more following intervention.

**Figure 3:**
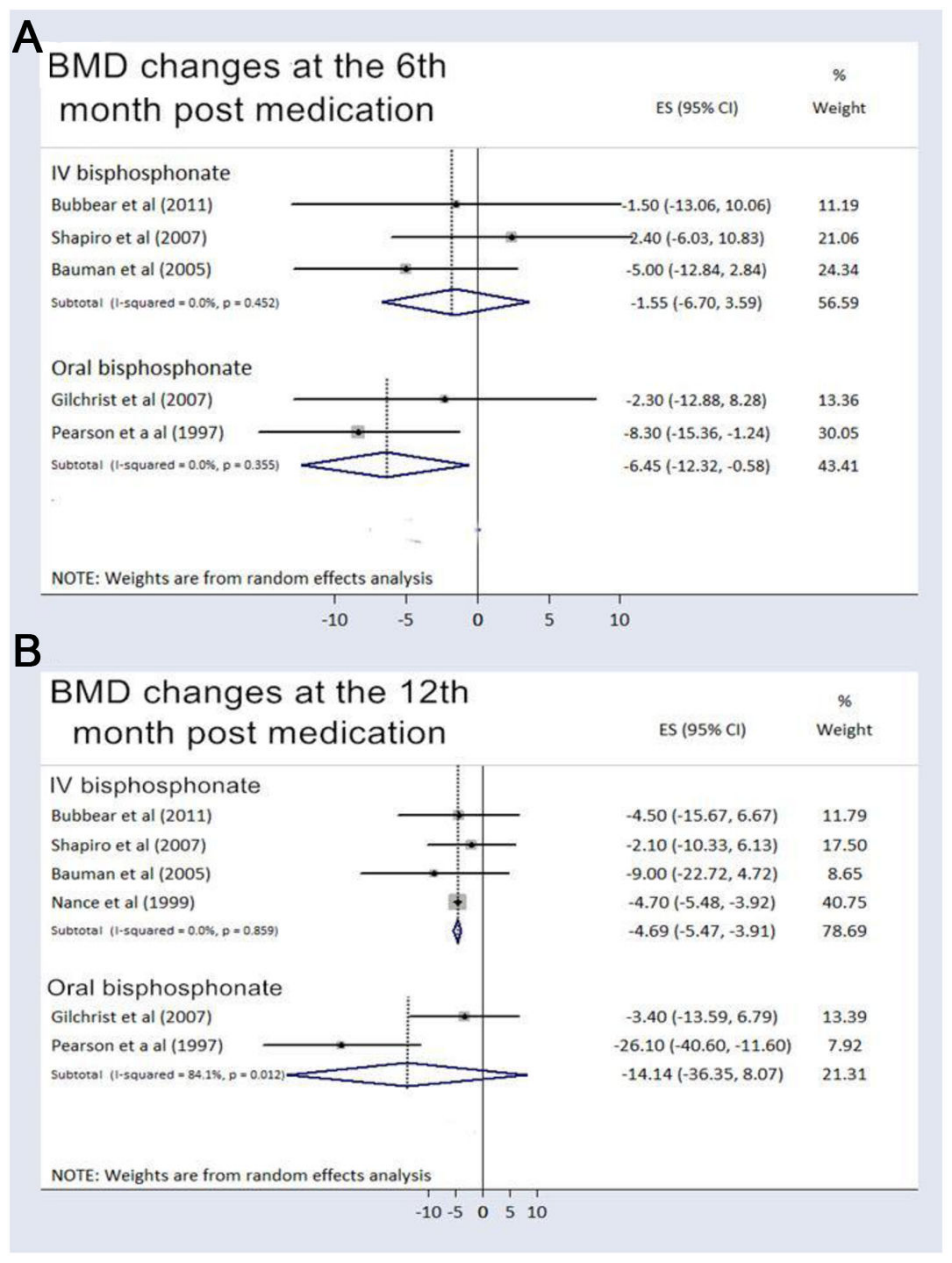
Percent bone mineral density changes categorized by the route of administration. The figure represents the forest plot of percent bone mineral density (BMD) change from baseline in the acute spinal cord injury patients using bisphosphonates categorized by the route of administration at (A) the 6th and (B) the 12th month following intervention.

For the acute SCI participants treated with bisphosphonates, the pooled BMD changes compared with baseline were -1.41% (95%CI, -4.86% to 2.05%) at the 3rd month, -3.68% (95% CI, -7.55% to 0.19%) at the 6th month, -6.11% (95% CI, -10.65% to -1.57% ) at the 12th month, and -9.85% (95% CI, -19.12% to -0.57%) at the 18th month or more after medication use. Among the control patients receiving placebo or usual care, the pooled BMD changes were -9.99% (95%CI, -13.04% to -6.94%) at the 3rd month, -9.99 % (95% CI, -18.25% to -1.73%) at the 6th month, -10.88% (95% CI, -11.66% to -10.10%) at the 12th month, and -21.93% (95% CI, -29.54% to -14.33%) at the 18th month or more after recruitment (Figure 2). If we eliminated Pearson’s study [19] that only prescribed bisphosphonates for two weeks at the beginning and end of the research, the pooled BMD changes increased to -1.70 % (95% CI, -6.32% to 2.93%) at the 6th month and -4.68% (95% CI, -5.46% to -3.91% ) at the 12th month. We performed a subgroup analysis for the route of bisphosphonate administration (Figure 3). The pooled BMD changes in trials using IV bisphosphonates were -1.55% (95% CI, -6.70% to 3.59%) at the 6th month and -4.69% (-95% CI, -5.47% to -3.91%) at the 12th month following treatments, compared with -6.45% (95% CI, -12.32% to -0.58%) at the 6th month and -14.14% (95% CI, -36.35% to 8.07%) at the 12th month with oral bisphosphonates (Figure 3). In the present meta-analysis, only two studies administered bisphosphonates in the chronic SCI population, whose pooled BMD change compared with baseline was 0.27% (95% CI, -0.85% to 1.39%) at the 6th month following bisphosphonate treatment.

**Figure 4:**
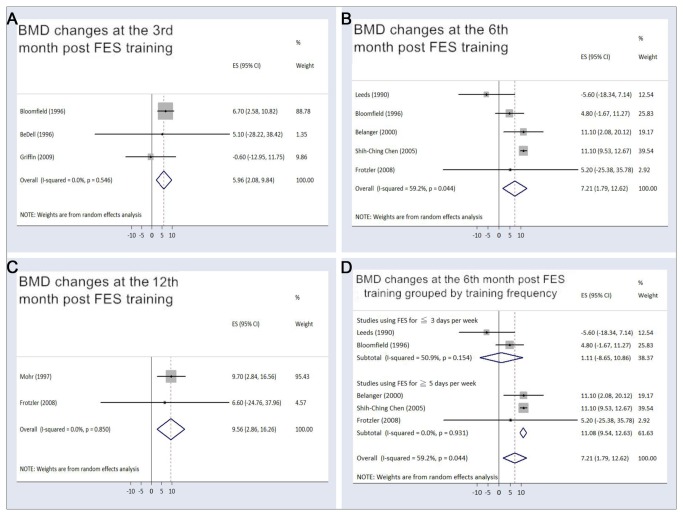
Percent bone mineral density changes in chronic spinal cord injury patients receiving functional electrical stimulation. The figure represents the forest plot of percent bone mineral density (BMD) changes from baseline in the chronic spinal cord injury patients receiving functional electrical stimulation (FES) training at (A) the 3rd, (B) the 6th, and (C) the 12th month following intervention; (D) forest plot of the percentage of bone mineral density (BMD) changes from baseline categorized by training frequency at the 6th month post-FES intervention.

**Figure 5:**
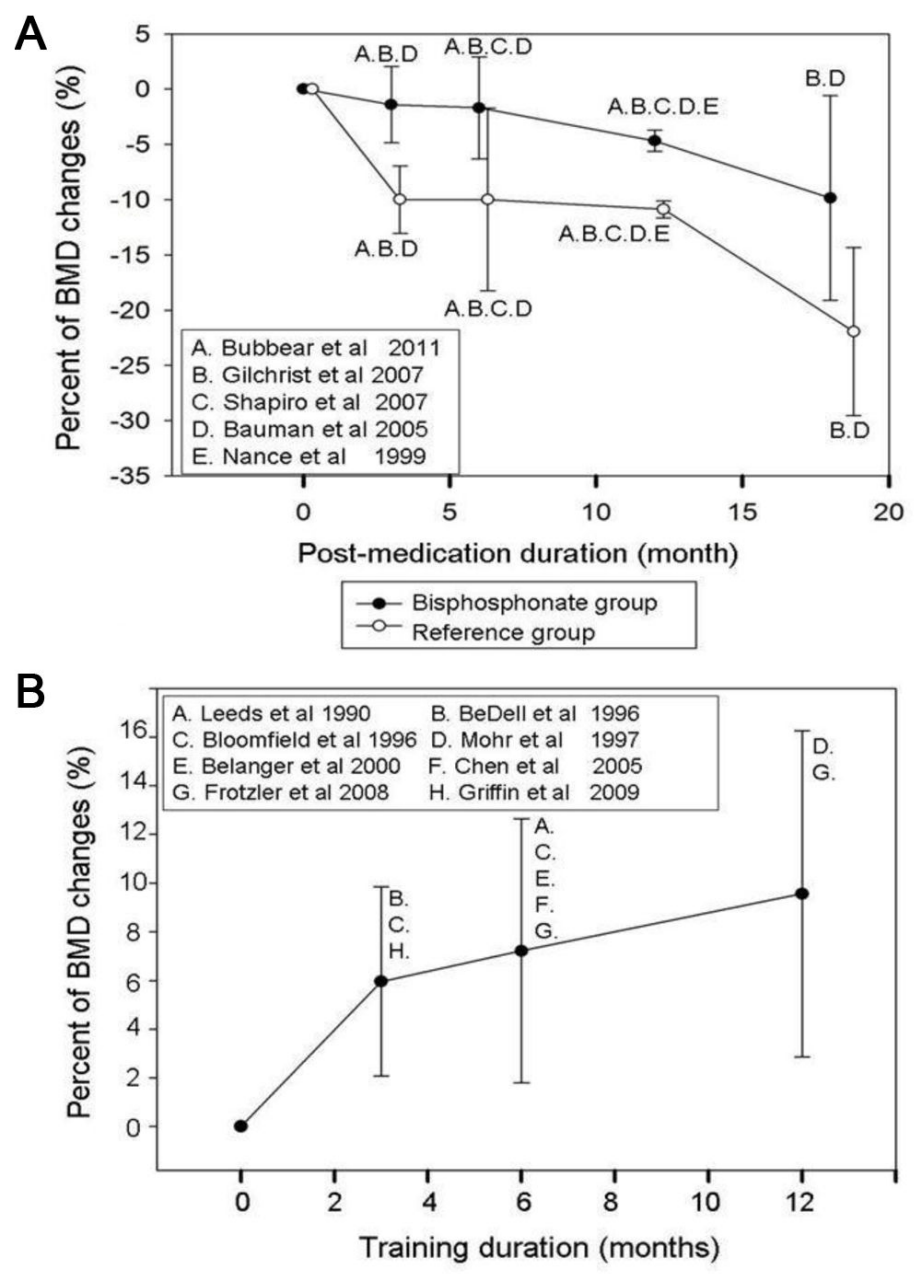
Temporal relationships of percent bone mineral density changes in studies employing bisphosphonates or functional electrical stimulation. The figure represents the temporal relationships of percent bone mineral density (BMD) changes from baseline in (A) studies prescribing bisphosphonates for acute spinal cord injury (SCI) patients and (B) trials using functional electrical stimulation (FES) for chronic SCI patients. The value was expressed by its pooled point estimate and 95% confidence interval.

Among the trials that applied FES on acute SCI patients, the pooled BMD changes from baseline were -1.89% (95% CI, -3.42% to -0.36%) at the 3rd month and -1.80% (95% CI, -8.86% to 5.26%) at the 6th month after intervention, whereas the values were -4.41% (95% CI, -7.90% to -0.91%) at the 3rd month and -4.58% (95% CI, -9.19% to 0.03%) at the 6th month in the participants receiving usual care. Among the studies using FES for the chronic SCI population, the pooled BMD changes from baseline were 5.96% (95% CI, 2.08% to 9.84%) at the 3rd month, 7.21% (95%CI, 1.79% to 12.62%) at the 6th month, and 9.56% (95% CI, 2.86% to 16.26%) at the 12th month following FES training. Regarding the influence of FES frequency on the effectiveness of training, a trend of toward higher elevation of BMD (11.08% [95% CI, 9.54% to 12.63%]), was identified in the studies using FES for at least 5 days per week at the 6th month following training. This finding is in stark contrast to the 1.11% (95% CI, -8.65% to 10.86) in the trials conducting FES ≤3 days per week (Figure 4). The temporal relationships of the BMD changes for acute SCI patients undergoing bisphosphonate therapy and chronic SCI patients receiving FES were illustrated in Figure 5.

### Safety

Among the 87 patients undergoing bisphosphonate therapy, 15 (17.2 %) patients experienced adverse effects. Eight patients receiving zoledronic acid reported flu-like symptoms such as myalgias and fever [24,25]. Other adverse events included gastrointestinal upset (n=4), dizziness (n=1), headache (n=1) after the usage of alendronate, and pruritic rash (n=1) following IV pamidronate. Among 149 patients who received FES, only one patient developed a foot fracture, which was unrelated to FES cycling training [38].

## Discussion

This meta-analysis identified 19 clinical trials prescribing bisphosphonate analogues or FES training for SCI patients and evaluated whether or not they could effectively ameliorate or reverse bone loss below the level of the SCI. The bisphosphonate group had less BMD decline than the control patients when the medication was initiated immediately after SCI. Moreover, FES training demonstrated an osteogenic effect on BMD near the distal femur in the chronic SCI group. Our review failed to prove an increase in sublesional BMD in chronic SCI patients using bisphosphonate or a decrease of BMD attenuation in acute SCI patients following FES training.

Two systematic reviews have investigated the utility of bisphosphonate analogues or rehabilitation-oriented methods for sublesional BMD in the SCI population. Bryson et al [8] identified 7 of the 8 bisphosphonate trials included in our meta-analysis and focused on criticisms of their methodology and evidence level. Prophylactic administration of bisphosphonates is not recommended by the authors based on the lower scores of research quality in retrieved studies and the absence of reporting fracture incidence. Biering-Sorensen et al [7] systematically reviewed the literature on non-pharmacologic approaches to treating osteoporosis following SCI and identified 9 of the 11 FES trials in our meta-analysis. A potential advantage of FES on BMD adjacent to the knee joint under a high-frequency and long-period training program was suggested. Both reviews bypassed quantitative analysis due to heterogeneity of the patient populations and outcome measures and were incapable of delineating the temporal relationship of BMD in groups receiving interventions compared with their pre-treatment values or controls. Therefore, we computed the percentage of change of BMD at different intervals, pooled their effect sizes for comparisons, and attempted to provide solid evidence regarding the influence of bisphosphonates or FES on sublesional BMD in SCI patients.

The present study indicated that the acute SCI population undergoing bisphosphonate therapy had less BMD reduction compared with the control patients. A significant superiority of intervention to reference treatments appeared at the 3rd month, and the trend was likely to be statistically significant at the 12th month based on a minimal overlap of the 95% CI of BMD changes between the intervention and control groups (Figure 2 and 5). Consequently, we performed a sensitivity analysis by eliminating Pearson’s study [19], which prescribed oral bisphosphonates for two weeks at the beginning and end of the research. The pooled percentage changes in BMD at the 12th month increased to -4.68% in the treatment population with a 95% CI (-5.46% to -3.91%) which separated from the corresponding value (-11.66% to -10.10%) in the reference group. The result confirmed the effectiveness of bisphosphonates and disclosed the importance of sustained bisphosphonate use in maintaining BMD for acute SCI participants. However, for chronic SCI participants, the pooled BMD change compared with baseline was 0.27% (95% CI, -0.85% to 1.39%) at the 6th month based on two studies using bisphosphonates [20,22]. Only one of the aforementioned studies followed the outcome for two years and found no significant increase in post-treatment BMD in chronic SCI individuals [20]. Therefore, current evidence did not prove an osteogenic effect of bisphosphonate administration in chronic SCI patients.

Some trials advocated the advantage of IV bisphosphonate over oral intake [21,24-26], but the comparison of the effectiveness for improving sublesional BMD has never been studied. IV bisphosphonate use eliminates the need to maintain an upright posture for the 30 minutes that is required with oral dosing, assures higher compliance with therapy, and provides better absorption without the interference of neurogenic bowel disorders in the SCI population [25]. Therefore, we performed a subgroup analysis comparing BMD changes between the use of IV and oral bisphosphonates in the acute SCI population. The crude data showed that bone loss in those patients receiving the IV regimen seemed to be less than in those patients taking oral bisphosphonates (Figure 3). However, if we excluded Pearson’s study [19] for its short use of medication, the point estimate of BMD changes from baseline in Gilchrist’s trial [23] was similar to the value in studies using IV administration (Figure 3). Since current oral bisphosphonates have proven to possess similar efficacy against osteoporotic hip fractures compared with the analogues administered via the IV route [10], our finding supported that the duration and not the route of bisphosphonate administration affected the BMD in acute SCI patients.

Regarding the effectiveness of FES, we found a significant increase in BMD in chronic SCI patients at the 3rd, 6th, and 12th months after intervention. Our results indicated that more than 3 months of FES training was capable of restoring BMD adjacent to the knee joint. Furthermore, a longer period of exercise could achieve better effectiveness (Figure 4 and 5). Since most FES studies used cycling ergometry or knee resistance exercises as their major functional task, the findings were compatible with the augmentation of BMD through FES exclusively occurring near the site directly exposed to mechanical loading, such as the distal femur and proximal tibia. Another concern was that the benefit of osteogenesis could not be maintained after FES was discontinued. According to Chen’s report [36], the BMD returned to the baseline value after 6 months without training, which stressed the importance of a continuous program for FES intervention.

We also conducted a subgroup analysis looking at the training frequency and BMD changes at the 6th month, which was the time interval with the most available BMD data (Figure 4). Our investigation demonstrated that studies using FES ≤3 days per week demonstrated only a pooled 1.11% increase in BMD (95% CI [-8.65% to 10.86]). In contrast, the trials employing FES ≥5 days per week had a pooled 11.08% elevation in BMD, which was statistically significant. This finding suggested 5 days per week should be the minimum training frequency in order to achieve significant improvement in BMD. Finally, compared with control patients, the present meta-analysis failed to show a benefit of FES in decreasing osteoporosis in the acute SCI population.

Our results suggested that the effectiveness of bisphosphonate administration and FES training were in accordance with the unique pathophysiology of osteoporosis in SCI patients. Osteoclastic activity overwhelms osteoblastic activity, which leads to rapid demineralization in the acute SCI following injury [2,43]. Therefore, it was reasonable that bisphosphonate, a potent inhibitor of osteoclast-mediated bone resorption, should help attenuate bone loss in acute SCI patients [8]. With chronic SCI, a steady state between osteogenic and osteolytic activity has been established and the benefit of bisphosphonates to inactivate osteoclasts became less significant [1]. FES training, which uses strenuous muscle contraction and mechanical loading to elicit osteogenesis, was shown to effectively restore BMD at the bony structures adjacent to the stimuli. Our meta-analysis implies that bisphosphonates should be administered from the beginning of the SCI and continued until a steady state has been reached (≈2 yr) in order to prevent loss of BMD. FES training can be used as an adjuvant tool to reverse muscle atrophy and activate bone formation in chronic, stable SCI patients.

Several limitations should be considered in the interpretation of the present meta-analysis. First, none of the studies had sufficient time to follow up subsequent fracture events. Although our findings demonstrated potential benefits of bisphosphonates or FES for improving sublesional BMD, some uncertainty remains regarding the effectiveness to reduce the rates of osteoporosis-related fractures. Secondly, most of the included FES studies used a pre-post test design without random assignment of enrolled participants or comparison with usual care. These fundamental flaws rendered those FES trials low in research quality and level of evidence. Thirdly, diversity of bisphosphonate dosage and protocols of administration existed across studies. The limitation was partly compensated by subgroup analysis based on the routes of administration and timings of BMD measurement. Besides, heterogeneity between trials usually biases the treatment effect toward a null result. However, our investigation demonstrated some significant results which made the effectiveness of both therapeutic approaches more convincing. Finally, most of the patients were males with diverse injury levels and post-injury durations. The heterogeneity with respect to injury level and duration of injury tended to reduce the precision of the outcome measurements and caused potentially insignificant results. Therefore, we analyzed the BMD changes separately in acute and chronic SCI populations and demonstrated some favorable results of both therapeutic approaches after compensating for bias from different post-injury periods. We should also be cautious about generalizing our study findings to female SCI patients.

In conclusion, the present meta-analysis revealed that bisphosphonate administration immediately after the SCI event was effective at attenuating the rate of bone loss below the SCI level. Sustained use of bisphosphonates until a steady state between osteogenic and osteolytic activities is reached is crucial in reducing osteoporosis in the acute SCI population. FES intervention could significantly increase sublesional BMD adjacent to the site of maximal mechanical loading in patients with chronic SCI, and training a minimum of 5 days per week was associated with higher effectiveness. 

## Supporting Information

Checklist S1 **PRISMA Checklist for systematic review and meta-analysis.**
(DOC)
